# Clonal Relatedness among Imipenem-Resistant* Pseudomonas aeruginosa* Isolated from ICU-Hospitalized Patients

**DOI:** 10.1155/2015/983207

**Published:** 2015-12-20

**Authors:** Hamid Vaez, Sharareh Moghim, Bahram Nasr Esfahani, Hajieh Ghasemian Safaei

**Affiliations:** Department of Microbiology, School of Medicine, Isfahan University of Medical Sciences, Isfahan, Iran

## Abstract

Imipenem-resistant* Pseudomonas aeruginosa* (*P. aeruginosa*) has become an increasingly important problem in healthcare settings worldwide. The aim of the present study was to evaluate clonal spread among imipenem-resistant* P. aeruginosa* isolated from ICU-hospitalized patients. Totally, 150 wound specimens were analyzed. Antibiotic resistance profiles and clonal diversity were evaluated using Kirby-Bauer's disk diffusion method and Random Amplified Polymorphic DNA- (RAPD-) PCR, respectively. The isolates showed a high frequency of antibiotic resistance against meropenem, and imipenem (100%) followed by ciprofloxacin, and ceftazidime (90%); meanwhile resistance to polymyxin B was not observed. Eighteen (40%) of* P. aeruginosa* isolates were MBL-positive via ethylenediaminetetraacetic acid (EDTA) combined disk test. Our findings showed high genetic diversity, with 37 different RAPD types detected. RAPD typing results showed cross-acquisition of* P. aeruginosa* in investigated hospital, suggesting failure in infection control practices. Incidence of MBL-positive isolates is high and should be regarded as a threat to hospitalized patients.

## 1. Introduction

*Pseudomonas aeruginosa* (*P. aeruginosa*) is a well-known hospital-acquired pathogen that has been frequently isolated from different kinds of infections such as wound infections, urinary tract infections (UTIs), respiratory tract infections, and blood stream infections (BSI), especially in intensive care unit (ICU) patients [1–3]. Since the antibiotic resistance in* P. aeruginosa* against different classes of antibiotics is growing rapidly, its treatment is becoming more challenging with each passing year [4].

Carbapenem antibiotics such as imipenem and meropenem were very effective in the treatment of* P. aeruginosa* infections, when first introduced; however, after less than decade of use resistance to these antibiotics by metallo-*β*-lactamase- (MBL-) producing strains emerged in clinical settings. A variety of mechanisms of resistance to carbapenems have been observed, including MBL-enzymes, efflux pumps overexpression, OprD mutations, and class D *β*-lactamases [5, 6].

Treatment of infections caused by imipenem-resistant* P. aeruginosa* is difficult, owing to the fact that imipenem resistance genes are usually located in transferable genetic elements such as plasmids and integrons along with other antibiotic resistant genes [7, 8]. These observations are consistent with the high mortality and morbidity rates observed among patients infected with imipenem-resistant* P. aeruginosa*. Moreover production of MBL-enzymes confers resistance to virtually all *β*-lactam antibiotics, except for monobactam [9].

Molecular epidemiological methods such as Random Amplified Polymorphic DNA- (RAPD-) PCR are well suited to determination of transmission routes of* P. aeruginosa* in hospital wards [10, 11]. An evaluation of the incidence of imipenem-resistant* P. aeruginosa* and its transmission in hospitals is of particular importance for the effective management of hospital-acquired infections. The objective of this study was to evaluate the incidence of imipenem-resistant* P. aeruginosa* in ICU-hospitalized patients and to document the potential for clonal spread.

## 2. Methods

### 2.1. Specimen Collection and Bacterial Identification

In total, 150 wound swabs obtained from burned patients admitted to the ICU ward of a referral hospital in Isfahan, between January 2013 and July 2014, were evaluated. Wound infection was identified based on clinical signs, described previously [12]. Only one isolate per patient was included in the study. The study was approved by the ethics committee of the Isfahan University of Medical Sciences (number 392063).

Presumptive identification of* P. aeruginosa* was performed by using standard conventional biochemical test including Gram-staining, catalase, oxidase, pigment production, oxidative-fermentative (OF) tests, and growth at 42°C [13]. Subsequently, species-specific PCR was done using previously designed primer for ITS (16s–23s rRNA internal transcribed spacer) [14]. PCR mixture (25 *μ*L) was made consisting of 200 *μ*M dNTPs, 2.5 mM MgCl_2_, PCR buffer 1x, Taq DNA pol 1.5 unit (Cinna Gen, Iran), 10 pM of each primer (Metabion, Germany), and 40 ng DNA sample. The following cycling conditions were applied: initial denaturation at 95°C 5 min, 1 cycle, 35 cycles consisting of denaturation at 94°C 1 min, annealing at 58°C 45 s, extension at 72°C 45 s, and final extension at 72°C for 5 min.

### 2.2. DNA Extraction

Bacterial fresh colonies (two or three) were removed and suspended in 300 mL of lysis buffer containing Tris 100 mM, Nacl 50 mM, and EDTA 25 mM, pH = 7.5, completely. Then suspension was boiled at 95°C for 15 min. Equal volume of phenol/chloroform (25 : 24, pH = 7.5) was added, mixed thoroughly, and centrifuged at 9000 g for 5 min. Aqueous-viscous supernatant was transformed to a new micro tube and phenol/chloroform (25 : 24) was added again and centrifuged for 5 min at 9000 g. 600 *μ*L cold and pure ethanol (Merck, Germany) was added and centrifuged at 13000 g, (4°C-30 min) to precipitate DNA. DNA washed twice in ethanol 70% and after quality check was stored at −20°C.

### 2.3. Antibiotic Susceptibility Test

Resistance to different antibiotics was tested by Kirby-Bauer's disk diffusion method based on CLSI (Clinical Laboratory Standard Institute) guidelines [15]. The following disks (MAST, UK) were applied: ceftazidime (CAZ, 30 *μ*g), imipenem (IMP, 10 *μ*g), meropenem (MEM, 10 *μ*g), ciprofloxacin (CIP, 5 *μ*g), aztreonam (ATM, 30 *μ*g), polymyxin B (PB, 300 units), and amikacin (AMK, 30 *μ*g).* P. aeruginosa* standard strain (ATCC 27853) was used as the quality control.

### 2.4. Detection of MBL

*P. aeruginosa* isolates that were resistant to imipenem and/or meropenem were subjected to phenotypical detection of MBL-producing isolates by imipenem-EDTA combined disk test, as described earlier [16]. Difference of ≥7 mm between the inhibition-zone diameter of the imipenem-EDTA disk and that of imipenem-only disk was considered as MBL-positive [16]. We used EDTA disk by itself and a strain of* P. aeruginosa* known to produce VIM-1 metallo-beta-lactamase as the negative and positive control, respectively.

### 2.5. Random Amplified Polymorphic DNA- (RAPD-) PCR

All* P. aeruginosa* isolates were submitted to RAPD-genotyping using primer 272-AGCGGGCCAA as previously described [17]. Briefly, optimized PCR mixture was made using 2.5 *μ*L 10x PCR buffer, 2.5 mM MgCl_2_, 300 *μ*M of dNTPs, 1.7 U Taq DNA polymerase (Cinna Gen, Iran), and 3 *μ*L genomic DNA (40 ng) in 25 *μ*L final volume. The following thermocycler program was used, (1) denaturation 5 min at 95°C, annealing 5 min at 36°C, and elongation 5 min at 72°C, for 4 cycles and (2) 31 cycles consisting of 94°C for 1 min, 45°C for 1 min, and 72°C for 2 min, followed by a final extension at 72°C for 10 min. To ensure reproducibility, each reaction was repeated three times. Similarity between isolates was evaluated based on Dice similarity coefficient and Unweighted Average Pair Group Method (UPGMA), using FreeTree and TreeView software [18, 19]. Only major reproducible bands regardless of intensity were considered for similarity matrix calculation [20] Cut-off value ≥80% was used for determination of potential clonal relatedness [21, 22].

## 3. Results

**Figure 1:**
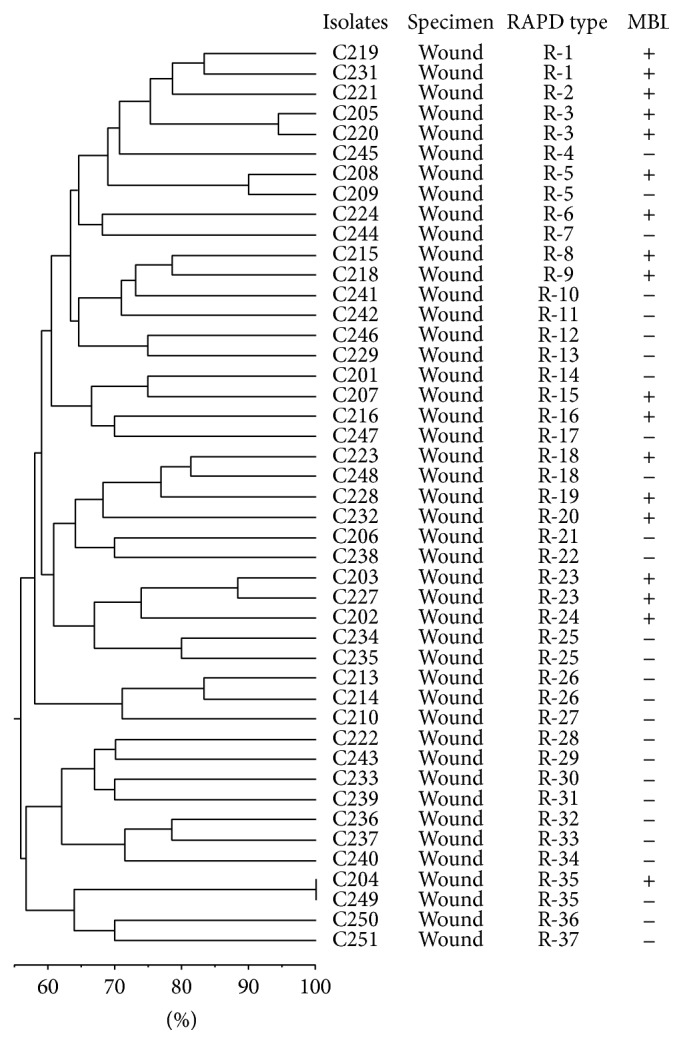
Dendrogram showing genetic diversity among 45 nonduplicate imipenem-resistant* P. aeruginosa* by RAPD-PCR.

Out of 150 tested specimens, 45 (30%) patients had positive culture for* P. aeruginosa*. Only one isolate per patient was recruited for study. Of the 45 patients, 30 (66%) were females and 15 (34%) were males. The isolates showed a high frequency of antibiotic resistance against meropenem, and imipenem (100%) followed by ciprofloxacin, aztreonam, and ceftazidime (90%), whereas the lowest resistance rate 61.6% was seen against amikacin. Resistance to polymyxin B was not observed. Eighteen (40%) of imipenem-resistant isolates were MBL-positive, using IMP-EDTA combined disk test. This study revealed high diversity of RAPD types, with 37 different RAPD types (Figure 1). Twenty-nine strains of* P. aeruginosa* showed unique patterns, while the rest of the strains (16) formed 8 distinct clusters. Groups one and three are composed of 2 isolates; these isolates were also MBL-positive.

## 4. Discussion

Hospitalized patients, particularly those who were admitted to the ICU, are mostly at risk for* P. aeruginosa* life-threatening infections [23]. Owing to the fact that imipenem resistance genes are usually located in the transposable genetic elements, along with other antibiotic resistance genes [24], the emergence of imipenem-resistant isolates of* P. aeruginosa* in healthcare settings is a serious problem.

In the present study, the highest percentages of resistant isolates were seen against aztreonam, meropenem, ceftazidime, and ciprofloxacin. Although this finding was supported by other studies carried out in Babol province (ceftazidime 92.6%) and Tehran province (imipenem and meropenem 94.7%, ceftazidime 89.4%, and ciprofloxacin 96.2%) of Iran, antibiotic resistance rates reported from Hamadan province (imipenem 7.5%, meropenem 13.2%, and ciprofloxacin 4.7%) were substantially lower than our resistance frequency [25–27]. In addition, our antibiotic resistance rates were significantly higher than those reported from some European countries [28–30]. This higher resistance rate probably resulted from indiscriminate use of antibiotics. According to independent study, presence of some risk factors, including use of catheter, ventilator, and previous consumption of antibiotics are associated with higher antibiotic resistance rates [31].

MBL was observed in 18 (40%) of the 45 imipenem-resistant isolates. This prevalence of MBL is much lower than that reported from other parts of Iran (Tehran province 94% and Zanjan province 87.8%) but is significantly higher than that reported from Sweden (1%) [32–34]. In MBL-negative isolates, probably other mechanisms such as OprD mutations, efflux pumps overexpression, or class D *β*-lactamase are responsible for carbapenems resistance [35]. In case of use of EDTA as MBL inhibitor due to inhibitory effect on bacterial growth, prevalence of MBL-positive isolates should be reported with precaution because it may lead to false-positive results.

Since determining bacterial genetic relatedness is essential for cross-infection evaluation, different genotyping methods have been established [36–39]. In spite of some of the limitations such as difficulty in interpretation of bands in some cases, RAPD-PCR have the advantage of being rapid, simple, and reproducible with high discriminatory power [36–39]. The RAPD-PCR fingerprinting technique yields reliable evaluations of clonal diversity [10, 40]. We detected the high genetic variability, with 37 distinct RAPD types among 45 isolates (82.2% of polymorphisms). Similarly, Silva reported 86 distinct RAPD types (89.6% of polymorphisms) among the 96 strains isolated from clinical specimens of different Brazilian hospitals [41]. In agreement with Pereira study in two referral hospitals of Portugal, our results did not reveal epidemic spread [11]. Our results demonstrate that most of the isolates probably originated from the patients themselves; however, cross-infection of* P. aeruginosa* between patients is possible to occur, suggesting nosocomial infection control problem.

## 5. Conclusion

According to our data incidence of imipenem-resistant isolates of* P. aeruginosa* is in alarming level. Our results did not demonstrate epidemic clone. Probable cross-acquisition has occurred and needs to be considered for future infection control procedure.
